# Association between impulsivity traits and body mass index at the observational and genetic epidemiology level

**DOI:** 10.1038/s41598-019-53922-8

**Published:** 2019-11-26

**Authors:** David Meyre, Sebat Mohamed, Joshua C. Gray, Jessica Weafer, James MacKillop, Harriet de Wit

**Affiliations:** 1 Department of Health Research Methods, Evidence, and Impact, McMaster University, Hamilton, Ontario Canada; 2 Department of Pathology and Molecular Medicine, McMaster University, Hamilton, Ontario Canada; 3 Department of Medical and Clinical Psychology, Uniformed Services University, Bethesda, Maryland United States of America; 4 Department of Psychiatry and Behavioral Neuroscience, University of Chicago, Chicago, Illinois United States of America; 5 Peter Boris Centre for Addictions Research, McMaster University/St. Joseph’s Healthcare Hamilton, Hamilton, Ontario Canada

**Keywords:** Genetic association study, Risk factors

## Abstract

We investigated the association between impulsivity related traits and BMI at the observational and genetic epidemiology level in a cross-sectional population of healthy young American-European adults. We studied 998 students and university staff of European ancestry recruited from Chicago (Illinois) and Athens (Georgia). We measured 14 impulsivity variables using three broad categories: impulsive choice, action and personality. Weight and height of participants were measured by research assistants. The single-nucleotide polymorphism (SNP) rs3751812 in the fat mass and obesity-associated (*FTO*) gene was genotyped using the Illumina PsychArray BeadChip platform. Within the three broad domains of impulsivity, 4 parameters (delay discounting of rewards area under the curve and average of k indexes, Conner’s continuous performance test, and negative urgency) were associated with BMI. The *FTO* rs3751812 minor allele T was associated with higher BMI. Of the 14 impulsivity variables, rs3751812 T was associated with more premeditation and perseverance, before and after adjusting for BMI. The association between *FTO* rs3751812 and BMI adjusted for premeditation remained significant, but disappeared after adjusting for perseverance and for both perseverance and premeditation traits. Our observational and genetic data indicate a complex pattern of association between impulsive behaviors and BMI in healthy young American-European adults.

## Introduction

Obesity is defined by the World Health Organization as an abnormal or excessive fat accumulation that presents a risk to health (https://www.who.int/topics/obesity/en/). The world health organization recommends classifying individuals with a body mass index (BMI) ≥30 kg/m^2^ as having obesity. The prevalence of obesity has been on the rise in the last four decades and is a global health concern^1^. Its rate is even more alarming in the United States (US) where adult prevalence of obesity reached 39.8% in 2015–2016, according to the Centers for Disease Control and Prevention (https://www.cdc.gov/obesity/index.html). Health complications associated with obesity (e.g. depression, sleep disorders, osteoarthritis, type 2 diabetes, fatty liver, hypertension, coronary artery disease and certain cancers) result in a decreased quality of life, decreased number of years adjusted for disability, and increased mortality^2,3^. In the US, the direct medical cost of excess adiposity combined was 5.0% to 10% of national healthcare spending in 2008^4^. Despite the availability of several therapeutic options (lifestyle and behavioral modifications, oral medication, bariatric surgery), obesity remains difficult to treat^5^. As obesity is a complex multifactorial disorder, research on its causes is expected to improve prediction, prevention and care in the long run^6^.

Obesity results from the interplay of environmental, societal, behavioral and biological, including genetic, influences^7^. Several human behaviors and personality traits have been associated with a risk for obesity (e.g. high neuroticism, low conscientiousness, lack of self-discipline)^8^, and we and others have recently shown a positive association between impulsivity and BMI^9^. In psychology, impulsivity is a tendency to act on a whim, displaying behavior characterized by little or no forethought, reflection, or consideration of the consequences^10^. Children, adolescents and adults from diverse ethnic groups displaying more impulse on delay discounting, sustained attention, and behavioral disinhibition are more likely to have obesity^9^. There is also evidence for an inherited component for impulsivity and obesity traits. For instance, twin studies provide heritability estimates of 69–75% for BMI and 46–62% for delay discounting^11,12^. While genome-wide association studies for delay discounting have only identified one SNP (rs6528024) in the intron of the gene Glycoprotein M6B so far^13^, over 940 polygenic loci associated with BMI/obesity have been reported in genome-wide association studies literature^14^. Among them, SNPs in intron 1 of the *FTO* gene are the more strongly associated with polygenic obesity in European, African and East Asian populations^12^. Two studies investigated the association of *FTO* SNPs with impulsivity traits in European adult populations, and reported inconsistent findings^15–17^. This prompted us to investigate the association of BMI and *FTO* rs3751812 SNP with 14 traits related to impulsivity in up to 998 healthy young adults of European ancestry residing in the US.

## Results

### Characteristics of the population

Table 1 contains a summary of the descriptive characteristics of the 998 students and staff from the University of Chicago and the University of Georgia included in this study. All participants are of European ancestry. Of these, 617 (61.8%) are women and 381 (38.2%) are men, with a mean age of 21.67 ± 3.31 years and mean BMI of 24.06 ± 4.61 kg/m^2^. People with underweight, normal weight, overweight and obesity represented 4.1%, 64.3%, 21.7% and 9.9% of the sample population, respectively. The genotype distribution of the *FTO* rs3751812 SNP is 33.1% GG (N = 330), 49.1% GT (N = 490) and 17.8% TT (N = 178). The rs3751812 T allele frequency was 42.4% in our sample, which was similar to the frequency reported in the European subpopulation of the 1000 Genomes Project (41.4%). A total of 14 impulsivity parameters grouped into three categories (impulsive choice, impulsive action, and impulsive personality) are outlined in Table 1.

**Table 1:** Participant characteristics, genotypes and impulsivity parameter.

Variable	% (N)/mean ± SD (N)
**Descriptive characteristics and genotypes**	
Ethnicity (European ancestry)	100% (998)
Sex (women/men)	61.8% (617)/38.2% (381)
Age	21.67 ± 3.31 (998)
BMI	24.06 ± 4.61 (993)
Underweight	4.1% (41)
Normal weight	64.3% (639)
Overweight	21.7% (215)
Obesity	9.9% (98)
*FTO* rs3751812	GG: 33.1% (330); GT: 49.1% (490); TT: 17.8% (178)
**Impulsive choice**	
Delay discounting of rewards single item yes/no	70.77% (702)/29.23% (290)
Task-based delay discounting of rewards area under the curve ($100 delayed reward)	0.56 ± 0.27 (992)
MCQ-based delay discounting of rewards: average of log transformation of k index for large, medium, and small items	−2.10 ± 0.64 (986)
**Impulsive action**	
Go/No Go Task: inhibition of a prepotent response	6.32 ± 2.76 (921)
Stop Signal Task: inhibition of a prepotent response errors	14.69 ± 7.36 (936)
Conner’s continuous performance test: inhibition of a prepotent response	15.43 ± 6.89 (949)
**Impulsive personality**	
UPPS-P: negative urgency	2.07 ± 0.50 (983)
UPPS-P: lack of premeditation	1.94 ± 0.42 (983)
UPPS-P: lack of perseverance	1.86 ± 0.44 (983)
UPPS-P: sensation seeking	2.80 ± 0.55 (983)
UPPS-P: positive urgency	1.59 ± 0.47 (983)
BIS: non-planning	22.10 ± 4.30 (991)
BIS: attentional subscale	16.57 ± 3.52 (991)
BIS: motor subscale	22.06 ± 3.84 (991)

MCQ: Monetary Choice Questionnaire; UPPS-P: UPPS-P Impulsive Behavior Scales; BIS: Barrett Impulsivity Scales. Data are mean ± standard deviation for continuous traits and numbers and percentages for categorical traits.

### Association of impulsivity traits with BMI

Table 2 presents a summary of the association of 14 impulsivity parameters with BMI, adjusted for sex, age and site. Within the three broad domains of impulsivity (impulsive choice, impulsive action, impulsive personality traits), 4 parameters were associated with BMI. From the impulsive choice domain, delay discounting of rewards area under the curve (Beta (B) = −0.021 ± 0.009, *P* = 0.018), delay discounting of rewards average of log transformation of k index for large, medium, and small items (B = 0.011 ± 0.004, *P* = 0.004) were associated with BMI. From the impulsive action domain, Conner’s continuous performance test (B = 0.0008 ± 0.0004, *P* = 0.027), and from the impulsive personality traits domain, negative urgency (B = 0.011 ± 0.005, *P* = 0.030) were associated with BMI. As delay discounting of rewards area under the curve has a reverse relationship to impulsivity, the direction of the effects for the 4 parameters is consistent with a higher BMI being associated with more impulsivity (Table 2).

**Table 2:** Association of impulsivity traits with BMI. Significant associations are bolded.

Variable	OR [95% C.I.] (*P*)/B ± SE (*P*)
**Impulsive choice**	
Delay discounting of rewards single item yes/no	0.470 [0.075–2.942] (0.420)
Task-based delay discounting of rewards area under the curve	**−0.021 ± 0.009 (0.018)**
MCQ-based delay discounting of rewards: average of log transformation of k index for large, medium, and small items	**0.011 ± 0.004 (0.004)**
**Impulsive action**	
Go/No Go Task: inhibition of a prepotent response	0.000286 ± 0.001 (0.745)
Stop Signal Task: inhibition of a prepotent response errors	−0.000129 ± 0.000254 (0.612)
Conner’s continuous performance test: inhibition of a prepotent response	**0.0008 ± 0.0004 (0.027)**
**Impulsive personality**	
UPPS-P: negative urgency	**0.011 ± 0.005 (0.030)**
UPPS-P: lack of premeditation	−0.002 ± 0.006 (0.668)
UPPS-P: lack of perseverance	−0.009 ± 0.005 (0.093)
UPPS-P: sensation seeking	−0.004 ± 0.004 (0.373)
UPPS-P: positive urgency	0.003 ± 0.005 (0.602)
BIS: non-planning	−0.001 ± 0.001 (0.179)
BIS: attentional subscale	−0.000192 ± 0.001 (0.783)
BIS: motor subscale	0.00008 ± 0.001 (0.895)

OR = odds ratio; CI = confidence interval; B = beta; SE = standard error; MCQ = Monetary Choice Questionnaire; UPPS-P = UPPS-P Impulsive Behavior Scales; BIS = Barrett Impulsivity Scales. Continuous traits: Data presented are β ± SE (*P*). Binary traits: Data presented are OR [95% C.I.] (*P*). Data was adjusted for sex, age and site.

### Association of *FTO* rs3751812 with BMI

The rs3751812 minor allele T was associated with a higher BMI (B = 0.007 ± 0.003, *P* = 0.03) in the young adult population.

### Association of *FTO* rs3751812 with impulsivity traits

Table 3 contains a summary of the association of rs3751812 SNP with 14 impulsivity traits adjusted for sex, age, and site. The same analysis adjusted for BMI was performed to test if BMI drives the association between *FTO* and impulsivity traits. The rs3751812 minor allele T was associated with 2 traits in the impulsive personality domain. These include lack of premeditation and lack of perseverance. When not adjusted for BMI, the *P* values were 0.009 (B = −0.050 ± 0.019) and 0.010 (B = −0.052 ± 0.020), respectively. When adjusted for BMI, the *P* values were 0.009 (B = −0.050 ± 0.019) and 0.017 (B = −0.048 ± 0.020), respectively. The direction of the effect was negative for both traits, meaning that the obesity risk allele is associated with more premeditation and more perseverance (Table 3). No association was found between rs3751812 and the remaining 12 traits, before and after adjusting for BMI.

**Table 3:** Association of *FTO* rs3751812 with impulsivity traits adjusted and unadjusted for BMI. Significant associations are bolded.

Variable	Β ± SE (adjusted *P*)/β ± SE (unadjusted *P*)
**Impulsive choice**	
Delay discounting of rewards single item yes/no	0.939 ± 0.102 (0.535)/0.939 ± 0.101 (0.533)
Task-based delay discounting of rewards area under the curve	0.006 ± 0.013 (0.626)/0.005 ± 0.013 (0.666)
MCQ-based delay discounting of rewards: average of log transformation of k index for large, medium, and small items	0.001 ± 0.029 (0.977)/0.002 ± 0.029 (0.933)
**Impulsive action**	
Go/No Go Task: inhibition of a prepotent response	−0.151 ± 0.133 (0.255)/−0.144 ± 0.132 (0.276)
Stop Signal Task: inhibition of a prepotent response errors	0.085 ± 0.453 (0.851)/0.131 ± 0.451 (0.772)
Conner’s continuous performance test: inhibition of a prepotent response	−0.449 ± 0.316 (0.155)/−0.346 ± 0.315 (0.272)
**Impulsive personality**	
UPPS-P: negative urgency	−0.027 ± 0.023 (0.226)/−0.021 ± 0.023 (0.359)
UPPS-P: lack of premeditation	**−0.050 ± 0.019 (0.009)**/−**0.050 ± 0.019 (0.009)**
UPPS-P: lack of perseverance	**−0.048 ± 0.020 (0.017)**/−**0.052 ± 0.020 (0.010)**
UPPS-P: sensation seeking	−0.021 ± 0.025 (0.397)/−0.021 ± 0.025 (0.394)
UPPS-P: positive urgency	−0.004 ± 0.021 (0.859)/0.003 ± 0.021 (0.902)
BIS: non-planning	−0.370 ± 0.199 (0.064)/−0.370 ± 0.198 (0.063)
BIS: attentional subscale	−0.119 ± 0.159 (0.455)/−0.100 ± 0.159 (0.530)
BIS: motor subscale	−0.189 ± 0.176 (0.284)/−0.182 ± 0.175 (0.298)

B = beta; SE = standard error; MCQ = Monetary Choice Questionnaire; UPPS-P = UPPS-P Impulsive Behavior Scales; BIS = Barrett Impulsivity Scales. Data presented are β ± SE (adjusted *P*)/β ± SE (unadjusted *P*). Data was adjusted for sex, age, site and BMI in certain analyses.

### Relationships between *FTO* rs3751812, impulsivity traits and BMI

We adjusted the association test between *FTO* rs3751812 and BMI for sex, age, site, lack of premeditation and lack of perseverance, separately and together. The aforementioned association between rs3751812 and BMI unadjusted for impulsive personality traits was B = 0.007 ± 0.003 (*P* = 0.03). The association between rs3751812 and BMI survived after adjusting for lack of premeditation (B = 0.007 ± 0.003, *P* = 0.04), but not for lack of perseverance (B = 0.007 ± 0.003, *P* = 0.06). The association did not remain significant after simultaneously adjusting for the two impulsive personality traits (B = 0.007 ± 0.003, *P* = 0.05).

## Discussion

In this study, we show that 4 out of 14 parameters of impulsive behaviors were significantly associated with BMI. These include parameters from all three major domains: impulsive choice, impulsive action, and impulsive personality traits. Associations were detected with delay discounting of future rewards, behavioral inhibition measured by Conner’s continuous performance test, and negative urgency, reflecting a person’s proneness to act out during periods of negative affect. All parameters were related to BMI such that more impulsivity was associated with higher BMI. We also show that the *FTO* rs3751812 minor allele T is positively associated with BMI and negatively associated with 2 impulsive personality traits (lack of premeditation and lack of perseverance), independently of BMI. The association between rs3751812 and BMI survives after adjusting for lack of premeditation, but not for lack of perseverance or both for lack of perseverance and lack of premeditation.

The associations between several impulsivity domains and BMI have been previously reported in the literature. Amlung and colleagues reported a meta-analysis of 39 independent studies showing a positive association between delay discounting of food and money and obesity^9^. Furthermore, cross-sectional and longitudinal studies linking BMI and reward responsiveness, sensation seeking, lack of perseverance, lack of premeditation, positive and negative urgency have been reported in children and adults from Europe and the US^8,18–21^.

Factors such as age, sex, food addiction, and genetics have been shown to mitigate the association between impulsivity and BMI^8,13,19,20^. Our study reports an association between rs3751812 and BMI in US university students of European ancestry. This association is plausible, as (i) Universities in North America represent an obesity-prone environment for young adults^22^; (ii) SNPs in *FTO* are strongly associated with polygenic obesity in children and adults of European descent^23^; (iii) SNPs in *FTO* interact with obesity-prone environments to promote obesity^24^; (iv) an association between weight gain and *FTO* rs9939609 SNP (in strong linkage disequilibrium with rs3751812 in populations of European ancestry) has been previously reported in university students from the United Kingdom^25^.

The *FTO* rs3751812 obesity risk T allele is also negatively associated with lack of premeditation and lack of perseverance in our study. In 697 US adults of European ancestry followed for up to 23 years, rs1421085 C minor allele showed an association with increased BMI and reduced medial prefrontal cortical function during aging^16^. Consistent with reduced brain function in regions intrinsic to impulse control and taste responsiveness, rs1421085 C allele carriers exhibited increased excitement seeking and intake of fatty foods^16^. Of note, rs1421085 is in perfect linkage disequilibrium with rs3751812 in the five ethnic groups from the 1000 Genomes Project. In contrast, Jonassaint and colleagues did not find any association between *FTO* SNPs and the cognitive, motor and non-planning components of the Barratt impulsivity scales in 1 085 and 677 females of European descent with anorexia nervosa and healthy weight control, respectively^15^. While more studies are needed to understand the precise contribution of *FTO* SNPs to impulsive behaviors, additional lines of evidences in the literature make the association biologically relevant. SNPs in intron 1 of *FTO* have been associated with differences of methylation and expression of *FTO* in blood and neurons, respectively^26,27^. FTO is a nucleic acid demethylase and is highly expressed in the brain^28^. Loss-of-function mutations in *FTO* result in an autosomal-recessive lethal syndrome characterized by growth retardation, microcephaly, severe psychomotor delay, functional brain deficits, and facial dysmorphism^29^. BMI-increasing alleles in *FTO* have been associated with lower brain, cortical gray matter, and nucleus accumbens volumes, reduced activity in brain areas important for emotion, impulse control and reward responsiveness, structural brain atrophy, reduced verbal fluency, increased loss of control episodes over eating, and decreased risk of stress/nervousness, alcohol dependence, depression, and suicide^30^. SNPs in intron 1 of *FTO* also regulate the expression of other important genes in the regulation of body weight (e.g*. RPGRIP1L, IRX3*) in neurons^26,31–33^. Considering the important role of *RPGRIP1L* in cilium function^26,31^, and the critical link between cilium function, hyperphagic obesity and cognition (e.g. Bardet-Biedl syndrome)^34^, it is tempting to speculate that changes in brain expression of *RPGRIP1L* induced by *FTO* SNPs may also have an impact on impulsive behaviors.

Adjustment for BMI did not remove the association between rs3751812 and lack of perseverance and premeditation, suggesting that the SNP may directly influence impulsive behaviors independent of BMI. In contrast, the association between rs3751812 and BMI disappeared after adjusting for lack of perseverance or lack of perseverance and premeditation parameters together. These results suggest that the association between *FTO* and BMI may involve changes in impulsive behaviors.

It is important to note that the 4 impulsivity traits associated with BMI differ from the two impulsive personality traits associated with rs3751812 SNP in our study. Moreover, impulsivity traits are positively associated with BMI, whereas rs3751812 T allele is associated with decreased impulsivity and increased BMI. A possible explanation for these counterintuitive observations is because *FTO* is involved in a specific molecular pathway^28^, while the association between BMI and impulsivity is an integration of all genetic and environmental influences linking both traits. Consistent with this view is the recent demonstration that the impulsivity trait delay discounting shares a substantial genetic overlap with BMI in populations of European ancestry (r_g_ = 0.18 ± 0.08, P = 8.9 × 10^−3^)^13^. These data suggest the involvement of multiple genes in linking impulsive behaviors to obesity. SNPs in *FTO* account for 1% of BMI variation in European populations^30^, and may represent as such one of the many genetic determinants linking the association between impulsivity traits and BMI^9,20,35^. Our data suggests that *FTO* SNPs may have a pleiotropic/BMI-independent association with two impulsivity traits (lack of perseverance and lack of premeditation). Supporting this hypothesis is recent evidence of a BMI-independent association between *FTO* SNPs and brain aging markers^30,36^.

This study has several strengths. It addresses a controversy caused by two inconsistent studies that have investigated the association of *FTO* SNPs with impulsivity^15,16^. In addition, we provide accurate measurements of BMI and very detailed assessments of the multidimensional construct of impulsivity. Finally, our study includes original analyses to model the pattern of association between *FTO* SNP, impulsivity and BMI. We also acknowledge that the current research has limitations. Our sample is modest by the standards of genetic association studies and focuses predominantly on US university students of European descent, limiting our ability to generalize our results to other populations. Another limitation is that our study is not longitudinal. The cross-sectional nature of this study precludes causal inferences to be made about the association between impulsivity traits and BMI. Finally, only one obesity SNP (*FTO* rs3751812) was studied, but there are now more than 940 independent SNPs that have been convincingly associated with BMI/obesity in recent literature^14^. Studying the association of a polygenic obesity risk score with impulsivity traits represents an exciting direction of research.

In conclusion, our observational and genetic data indicate a complex pattern of association between impulsive behaviors and BMI in healthy young American-European adults. More research is needed to precise the contribution of SNPs in *FTO* and other obesity genes to impulsive behaviors in diverse ethnicities. Mendelian randomization studies are also warranted to evidence the causal nature of the association between impulsive behaviors and susceptibility to obesity.

## Methods

### Participants

Participants were recruited from the general community at the University of Chicago and the University of Georgia and from undergraduate programs at the University of Georgia. Participants were between 18 and 30 years old. They were excluded if they had positive breath alcohol tests or urine drug screen, or scores over 12 on the Alcohol Use Disorder Identification Test and Drug Use Disorder Identification Test, as previously reported^37^. The rationale for the drug-related eligibility was to avoid impulsivity phenotypes that could be a result of recent or significant habitual substance use^38^. Nine-hundred and ninety-eight unrelated young adults of European ancestry were included in the present analysis. Self-reported ethnicity was confirmed using ancestral informative markers in the principal component analysis EIGENSTRAT^39^. Additional details of participants can be found in a previous publication^37^. The study protocol was approved by the institutional review boards of the University of Chicago and the University of Georgia. Written informed consent was obtained from each subject prior to enrolment into the study. All procedures were conducted in accordance with the relevant guidelines and regulations of the Declaration of Helsinki^40^.

### Phenotyping

Weight (without shoes) and height of participants were measured by research assistants using the Taylor body fat scale (Taylor Precision Products, Las Cruces, New Mexico, USA). BMI was calculated as weight (kg) divided by height squared (m). Underweight, normal weight, overweight and obesity categories were defined according to the World Health Organization.

Impulsivity was measured using tasks from three broad categories^37^: (1) impulsive choice, also referred to as discounting of delayed rewards, was assessed via a monetary choice questionnaire^41^ and a delay discounting task^42^; (2) impulsive action, measuring the inhibition of a prepotent response, was assessed via a go/nogo task^43^, a stop signal task^44^ and a Conner’s continuous performance test^45^; and (3) impulsive personality was assessed by the UPPS-P Impulsive Scale^46,47^ for sensation seeking, lack of perseverance, lack of premeditation, positive urgency, negative urgency, and by the Barratt Impulsiveness Scale version 11^48^ for non-planning, attentional and motor traits. For the discounting of delayed rewards assessments, a criterion of 80% correct on the control items was used to define valid performance. For the behavioral inhibition tasks, invalid performance was defined as ≤80% accuracy on Go trials or ≥90% inhibitory errors, putatively reflecting misunderstanding of the correct response keys or very low task effort. Of note, the monetary delay discounting tasks were consequated for participants to receive the actual outcome of one of their choice using the approach developed for the Monetary Choice Questionnaire^41^. Additional details on the measurements of impulsivity can be found in a previous publication^37^.

### Genotyping

DNA was collected via a saliva sample for DNA collection in an Oragene DNA kit (DNA Genotek Inc., Kanata, ON, Canada). Genotyping was performed using the Illumina PsychArray BeadChip platform, which characterizes ~600 000 SNPs and has been optimized to capture the maximum amount of information about common variation. More details about the genotyping procedure have been previously published^49^. We focused our genetic analysis on the *FTO* locus as (i) it accounts for 1% of BMI variation and is the more important contributor to polygenic obesity in European populations^50^; (ii) several papers on its association with impulsivity traits have been published with inconsistent results^15–17^; (iii) associations between *FTO* SNPs and other psychological/psychiatric traits (e.g. loss of control episodes over eating, stress/nervousness, alcohol dependence, depression, suicide) have been reported in literature^30^; (iv) FTO is highly expressed in brain and has an essential role in brain development^29,51,52^; (v) a significant genetic correlation between BMI and impulsivity traits has been recently reported in populations of European ancestry^13^. An independent search of literature by S.M. and D.M. identified a list of 17 SNPs in *FTO* that reached a genome-wide significant association (*P* < 5 × 10^−8^) with BMI or binary obesity status in populations of European ancestry (rs9939609, rs9930506, rs9941349, rs7185735, rs11075990, rs3751812, rs8050136, rs8043757, rs17817449, rs1121980, rs55872725, rs9930506, rs1421085, rs9940128, rs9930333, rs9936385, rs1558902). We used additional criteria to select an optimal *FTO* SNP for our study: (i) SNP included in the PsychArray; (ii) SNP call rates > 0.97; (iii) SNP in Hardy-Weinberg equilibrium (P ≥ 0.0005); (iv) evidence that the SNP may be causal (based on trans-ethnic fine-mapping and functional characterization studies)^23,53,54^. Based on these criteria, we selected the SNP rs3751812 (genotype count: 328 GG, 488 GT, 175 TT; genotyping call rate = 0.993; P _Hardy-Weinberg_ = 0.78).

### Statistical analyses

Statistical analyses were performed using SPSS (version 20, New York, USA, IBM Corporation). We coded the rs3751812 GG, GT and TT genotypes as 0, 1 and 2, based on the number of copies of the BMI increasing/obesity risk alleles (T: effect allele; G: other allele). All genetic association studies were performed under an additive mode of inheritance, as *FTO* intron 1 SNPs have been consistently found to be the more important genetic contributors to BMI variation/obesity risk in populations of European ancestry under this model^50,55^. The association of clinical or genetic variables with binary and continuous outcomes was tested using logistic and linear regression models, respectively. Regression models were adjusted for covariates including age, sex, site, and impulsivity or BMI in certain analyses. The normal distribution of continuous variables was tested using the Shapiro-Wilk test. It is well-established that BMI distribution is skewed towards higher values in the general population. Logarithmic transformations were used to correct for the lack of normality of BMI values. Considering the correlation among impulsivity traits, and the prior likelihood of association between BMI and impulsivity traits^9,20,35^, *FTO* SNPs and BMI^23^, and *FTO* SNPs and impulsivity traits^15,16^, we considered a two-sided *P* < 0.05 as significant in this study.

## Data Availability

The dataset generated during and/or analyzed during the current study are available from the corresponding authors on reasonable request.

## References

[1] Ng M (2014). Global, regional, and national prevalence of overweight and obesity in children and adults during 1980-2013: a systematic analysis for the Global Burden of Disease Study 2013. Lancet.

[2] Fontaine KR, Redden DT, Wang C, Westfall AO, Allison DB (2003). Years of life lost due to obesity. Jama.

[3] Dixon JB (2010). The effect of obesity on health outcomes. Molecular and cellular endocrinology.

[4] Tsai AG, Williamson DF, Glick HA (2011). Direct medical cost of overweight and obesity in the USA: a quantitative systematic review. Obesity reviews: an official journal of the International Association for the Study of Obesity.

[5] Peirson L (2014). Treatment for overweight and obesity in adult populations: a systematic review and meta-analysis. CMAJ open.

[6] Pigeyre M, Yazdi FT, Kaur Y, Meyre D (2016). Recent progress in genetics, epigenetics and metagenomics unveils the pathophysiology of human obesity. Clin Sci (Lond).

[7] Reddon H, Gueant JL, Meyre D (2016). The importance of gene-environment interactions in human obesity. Clin Sci (Lond).

[8] Sutin AR, Ferrucci L, Zonderman AB, Terracciano A (2011). Personality and obesity across the adult life span. Journal of personality and social psychology.

[9] Amlung M, Petker T, Jackson J, Balodis I, MacKillop J (2016). Steep discounting of delayed monetary and food rewards in obesity: a meta-analysis. Psychological medicine.

[10] Bakhshani NM (2014). Impulsivity: a predisposition toward risky behaviors. International journal of high risk behaviors & addiction.

[11] Anokhin AP, Grant JD, Mulligan RC, Heath AC (2015). The genetics of impulsivity: evidence for the heritability of delay discounting. Biological psychiatry.

[12] Stryjecki C, Alyass A, Meyre D (2018). Ethnic and population differences in the genetic predisposition to human obesity. Obesity reviews: an official journal of the International Association for the Study of Obesity.

[13] Sanchez-Roige S (2018). Genome-wide association study of delay discounting in 23,217 adult research participants of European ancestry. Nature neuroscience.

[14] Yengo L (2018). Meta-analysis of genome-wide association studies for height and body mass index in approximately 700000 individuals of European ancestry. Human molecular genetics.

[15] Jonassaint CR (2011). Absence of association between specific common variants of the obesity-related FTO gene and psychological and behavioral eating disorder phenotypes. American journal of medical genetics. Part B, Neuropsychiatric genetics: the official publication of the International Society of Psychiatric Genetics.

[16] Chuang YF (2015). FTO genotype and aging: pleiotropic longitudinal effects on adiposity, brain function, impulsivity and diet. Molecular psychiatry.

[17] Wiemerslage L (2016). An obesity-associated risk allele within the FTO gene affects human brain activity for areas important for emotion, impulse control and reward in response to food images. The European journal of neuroscience.

[18] van den Berg L (2011). Association between impulsivity, reward responsiveness and body mass index in children. Int J Obes (Lond).

[19] Murphy CM, Stojek MK, MacKillop J (2014). Interrelationships among impulsive personality traits, food addiction, and Body Mass Index. Appetite.

[20] VanderBroek-Stice L, Stojek MK, Beach SR, vanDellen MR, MacKillop J (2017). Multidimensional assessment of impulsivity in relation to obesity and food addiction. Appetite.

[21] Mobbs O, Crepin C, Thiery C, Golay A, Van der Linden M (2010). Obesity and the four facets of impulsivity. Patient education and counseling.

[22] Morassut RE (2017). Rationale and design of GENEiUS: a prospective observational study on the genetic and environmental determinants of body mass index evolution in Canadian undergraduate students. BMJ open.

[23] Dina C (2007). Variation in FTO contributes to childhood obesity and severe adult obesity. Nature genetics.

[24] Abadi A (2017). Penetrance of Polygenic Obesity Susceptibility Loci across the Body Mass Index Distribution. American journal of human genetics.

[25] Meisel SF, Beeken RJ, van Jaarsveld CH, Wardle J (2015). The Association of FTO SNP rs9939609 with Weight Gain at University. Obesity facts.

[26] Stratigopoulos G (2016). Hypomorphism of Fto and Rpgrip1l causes obesity in mice. The Journal of clinical investigation.

[27] Bell CG (2010). Integrated genetic and epigenetic analysis identifies haplotype-specific methylation in the FTO type 2 diabetes and obesity susceptibility locus. PloS one.

[28] Gerken T (2007). The obesity-associated FTO gene encodes a 2-oxoglutarate-dependent nucleic acid demethylase. Science.

[29] Boissel S (2009). Loss-of-function mutation in the dioxygenase-encoding FTO gene causes severe growth retardation and multiple malformations. American journal of human genetics.

[30] Tung YCL, Yeo GSH, O’Rahilly S, Coll AP, Obesity (2014). and FTO: Changing Focus at a Complex Locus. Cell metabolism.

[31] Stratigopoulos G (2014). Hypomorphism for RPGRIP1L, a ciliary gene vicinal to the FTO locus, causes increased adiposity in mice. Cell metabolism.

[32] Smemo S (2014). Obesity-associated variants within FTO form long-range functional connections with IRX3. Nature.

[33] de Araujo TM (2019). The partial inhibition of hypothalamic IRX3 exacerbates obesity. EBioMedicine.

[34] Kaur Y, de Souza RJ, Gibson WT, Meyre D (2017). A systematic review of genetic syndromes with obesity. Obesity reviews: an official journal of the International Association for the Study of Obesity.

[35] Fields SA, Sabet M, Reynolds B (2013). Dimensions of impulsive behavior in obese, overweight, and healthy-weight adolescents. Appetite.

[36] Ganeff IMM, Bos MM, van Heemst D, Noordam R (2019). BMI-associated gene variants in FTO and cardiometabolic and brain disease: obesity or pleiotropy?. Physiological genomics.

[37] MacKillop J (2016). The latent structure of impulsivity: impulsive choice, impulsive action, and impulsive personality traits. Psychopharmacology.

[38] Quinn PD, Harden KP (2013). Differential changes in impulsivity and sensation seeking and the escalation of substance use from adolescence to early adulthood. Development and psychopathology.

[39] Price AL (2006). Principal components analysis corrects for stratification in genome-wide association studies. Nature genetics.

[40] World Medical Association Declaration of Helsinki: ethical principles for medical research involving human subjects. *Jama* **310**, 2191–2194, 10.1001/jama.2013.281053 (2013). 10.1001/jama.2013.281053 24141714

[41] Kirby KN, Petry NM, Bickel WK (1999). Heroin addicts have higher discount rates for delayed rewards than non-drug-using controls. Journal of experimental psychology. General.

[42] Amlung M, Sweet LH, Acker J, Brown CL, MacKillop J (2014). Dissociable brain signatures of choice conflict and immediate reward preferences in alcohol use disorders. Addiction biology.

[43] Kiehl KA, Laurens KR, Duty TL, Forster BB, Liddle PF (2001). Neural sources involved in auditory target detection and novelty processing: an event-related fMRI study. Psychophysiology.

[44] Verbruggen F, Logan GD (2008). Response inhibition in the stop-signal paradigm. Trends in cognitive sciences.

[45] Beck LH, Bransome ED, Mirsky AF, Rosvold HE, Sarason I (1956). A continuous performance test of brain damage. Journal of consulting psychology.

[46] Cyders MA (2007). Integration of impulsivity and positive mood to predict risky behavior: development and validation of a measure of positive urgency. Psychological assessment.

[47] Whiteside SP, Lynam DR (2001). The five factor model and impulsivity: using a structural model of personality to understand impulsivity. Personal Individ Differ.

[48] Patton JH, Stanford MS, Barratt ES (1995). Factor structure of the Barratt impulsiveness scale. Journal of clinical psychology.

[49] Gray JC (2018). Genetic analysis of impulsive personality traits: Examination of a priori candidates and genome-wide variation. Psychiatry research.

[50] Meyre D (2009). Genome-wide association study for early-onset and morbid adult obesity identifies three new risk loci in European populations. Nature genetics.

[51] Ho AJ (2010). A commonly carried allele of the obesity-related FTO gene is associated with reduced brain volume in the healthy elderly. Proceedings of the National Academy of Sciences of the United States of America.

[52] Melka MG (2013). FTO, obesity and the adolescent brain. Human molecular genetics.

[53] Grant SF (2008). Association analysis of the FTO gene with obesity in children of Caucasian and African ancestry reveals a common tagging SNP. PloS one.

[54] Hassanein MT (2010). Fine mapping of the association with obesity at the FTO locus in African-derived populations. Human molecular genetics.

[55] Scuteri A (2007). Genome-Wide Association Scan Shows Genetic Variants in the FTO Gene Are Associated with Obesity-Related Traits. PLoS genetics.

